# Supplementary material for: Trends, district-level variations, and socioeconomic disparities in cesarean section delivery in Bangladesh
Source: PLoS One. 2025 Oct 31;20(10):e0334931. doi: 10.1371/journal.pone.0334931 (PMC12578250; doi:10.1371/journal.pone.0334931)
Supplement: S1 Table — (DOCX) [file pone.0334931.s001.docx]

**Supplementary table 1: Background characteristics of the study respondents, 1999/2000 - 2022**

| **Characteristics** | **1999/2000,**  **% (95% CI)** | **2004,**  **% (95% CI)** | **2007,**  **% (95% CI)** | **2011,**  **% (95% CI)** | **2014,**  **% (95% CI)** | **2017/2018,**  **% (95% CI)** | **2022,**  **% (95% CI)** |
| --- | --- | --- | --- | --- | --- | --- | --- |
| **Mother’s age at birth, mean (±SD)** | 23.3 (±6.2) | 23.1 (±6.1) | 23.0 (±5.8) | 22.9 (±5.5) | 23.1 (±5.7) | 24.8 (±5.5) | 24.2 (±5.6) |
| **≤**19 years | 32.6 (30.8-34.5) | 32.8 (31.0-34.6) | 32.7 (30.9-34.6) | 31.1 (29.5-32.6) | 31.9 (30.1-33.6) | 29.6 (28.1-31.0) | 23.9 (22.6-25.2) |
| 20-34 years | 62.1 (60.3-63.9) | 61.7 (59.8-63.5) | 62.5 (60.6-64.3) | 65.2 (63.7-66.8) | 64.1 (62.3-65.9) | 67.0 (65.5-68.5) | 71.3 (69.9-72.7) |
| ≥35 years | 5.3 (4.5-6.2) | 5.5 (4.7-6.4) | 4.8 (4.0-5.8) | 3.7 (3.1-4.4) | 4.0 (3.2-5.0) | 3.4 (3.0-4.0) | 4.8 (4.2-5.5) |
| **Mother’s education** |  |  |  |  |  |  |  |
| No education | 45.9 (43.4-48.5) | 35.5 (33.0-38.0) | 23.9 (21.5-26.4) | 18.0 (16.1-20.0) | 14.4 (12.3-16.6) | 6.6 (5.7-7.6) | 5.4 (4.6-6.3) |
| Primary | 28.6 (27.0-30.3) | 30.6 (28.8-32.4) | 31.0 (28.9-33.2) | 30.0 (28.1-31.9) | 28.1 (26.2-30.1) | 27.6 (25.8-29.5) | 22.8 (21.3-24.4) |
| Secondary | 21.6 (19.8-23.6) | 28.3 (26.2-30.5) | 38.6 (36.1-41.2) | 44.6 (42.1-47.2) | 47.5 (44.8-50.3) | 48.8 (46.9-50.6) | 54.2 (52.4-55.9) |
| Higher | 3.9 (3.2-4.7) | 5.6 (4.8-6.7) | 6.5 (5.5-7.7) | 7.4 (6.5-8.5) | 10.0 (8.8-11.4) | 17.0 (15.5-18.6) | 17.6 (16.1-19.2) |
| **Mother’s formal employment status** |  |  |  |  |  |  |  |
| Employed | 16.0 (14.3-17.9) | 15.9 (14.1-17.9) | 23.0 (20.6-25.6) | 7.7 (6.7-8.8) | 23.7 (21.6-25.9) | 37.0 (34.8-39.2) | 22.1 (20.5-23.8) |
| Unemployed | 84.0 (82.1-85.7) | 84.1 (82.1-86.0) | 77.0 (74.4-79.4) | 92.3 (91.2-93.3) | 76.3 (74.1-78.4) | 63.0 (60.8-65.2) | 77.9 (76.2-79.5) |
| **Parity** |  |  |  |  |  |  |  |
| 1-2 | 53.4 (51.5-55.4) | 52.7 (50.5-54.9) | 60.3 (58.1-62.4) | 64.4 (62.4-66.3) | 68.7 (66.2-71.0) | 70.0 (68.3-71.6) | 70.6 (69.0-72.2) |
| >2 | 46.6 (44.6-48.5) | 47.3 (45.1-49.5) | 39.7 (37.6-41.9) | 35.6 (33.7-37.6) | 31.3 (29.0-33.8) | 30.0 (28.4-31.7) | 29.4 (27.9-31.1) |
| **Sex of child** |  |  |  |  |  |  |  |
| Male | 50.9 (49.3-52.4) | 50.1 (48.3-51.9) | 50.1 (48.1-52.1) | 50.6 (48.9-52.3) | 52.5 (50.5-54.5) | 52.4 (51.0-53.9) | 51.2 (49.7-52.7) |
| Female | 49.1 (47.6-50.7) | 49.9 (48.2-51.7) | 49.9 (47.9-51.9) | 49.4 (47.7-51.1) | 47.5 (45.5-49.5) | 47.6 (46.1-49.0) | 48.8 (47.3-50.3) |
| **Exposure to mass media** |  |  |  |  |  |  |  |
| Not exposed | 56.8 (54.1-59.4) | 32.4 (29.7-35.2) | 37.6 (34.8-40.6) | 36.5 (34.1-39.0) | 38.5 (35.6-41.5) | 34.8 (32.3-37.3) | 43.8 (41.4-46.0) |
| Moderately exposed | 39.9 (37.5-42.3) | 22.8 (21.2-24.4) | 22.3 (20.6-24.0) | 48.3 (46.0-50.5) | 48.0 (45.4-50.7) | 54.7 (52.3-57.0) | 50.8 (48.6-53.1) |
| Highly exposed | 3.3 (2.7-4.2) | 44.8 (42.2-47.4) | 40.1 (37.5-42.7) | 15.2 (13.9-16.7) | 13.5 (12.1-15.0) | 10.6 (9.5-11.8) | 5.4 (4.7-6.3) |
| **Wealth quintile** |  |  |  |  |  |  |  |
| Poorest | - | 25.0 (22.5-27.6) | 21.1 (18.7-23.8) | 22.9 (20.6-25.4) | 22.1 (19.4-25.1) | 20.8 (18.6-23.1) | 20.6 (18.9-22.5) |
| Poorer | - | 20.2 (18.4-22.0) | 21.7 (20.0-23.7) | 20.2 (18.7-21.9) | 19.0 (17.3-20.9) | 20.7 (19.2-22.4) | 20.9 (19.4-22.6) |
| Middle | - | 20.6 (18.9-22.4) | 19.5 (17.5-21.6) | 19.7 (18.1-21.3) | 19.2 (17.0-21.7) | 19.1 (17.6-20.8) | 20.7 (19.1-22.3) |
| Richer | - | 17.5 (15.8-19.3) | 19.3 (17.4-21.5) | 19.4 (17.8-21.1) | 20.3 (18.2-22.5) | 20.1 (18.3-22.0) | 19.8 (18.2-21.6) |
| Richest | - | 16.8 (14.9-19.0) | 18.4 (16.2-20.8) | 17.8 (16.1-19.6) | 19.4 (16.9-22.2) | 19.3 (17.4-21.4) | 18.0 (16.1-20.1) |
| **Place of residence** |  |  |  |  |  |  |  |
| Urban | 16.4 (14.9-17.9) | 19.8 (18.1-21.6) | 21.2 (19.6-22.9) | 22.6 (21.2-24.1) | 25.8 (23.1-28.7) | 26.7 (25.0-28.4) | 26.8 (24.9-28.7) |
| Rural | 83.6 (82.1-85.1) | 80.2 (78.4-81.9) | 78.8 (77.1-80.4) | 77.4 (75.9-78.8) | 74.2 (71.3-76.9) | 73.3 (71.6-75.0) | 73.2 (71.3-75.1) |
| **Division** |  |  |  |  |  |  |  |
| Barishal | 6.2 (5.3-7.1) | 5.8 (4.8-6.8) | 6.1 (5.4-6.9) | 5.5 (5.0-6.1) | 5.7 (4.8-6.8) | 5.7 (5.1-6.3) | 6.1 (5.4-6.8) |
| Chattogram | 22.4 (20.2-24.7) | 22.3 (20.7-24.1) | 22.2 (20.3-24.3) | 23.7 (22.1-25.4) | 21.9 (19.4-24.7) | 21.4 (19.7-23.2) | 22.2 (20.1-24.4) |
| Dhaka | 30.3 (28.2-32.5) | 30.3 (28.3-32.4) | 31.6 (29.4-33.8) | 30.5 (28.7-32.3) | 35.5 (31.1-40.1) | 25.6 (23.8-27.5) | 24.6 (22.8-26.6) |
| Khulna | 10.0 (9.0-11.3) | 10.9 (9.9-12.0) | 9.1 (8.1-10.3) | 9.3 (8.5-10.2) | 7.9 (7.0-8.9) | 9.0 (8.2-10.0) | 10.1 (9.2-11.1) |
| Mymensingh | - | - | - | - | - | 8.5 (7.6-9.4) | 8.8 (8.0-9.6) |
| Rajshahi | 23.6 (21.5-25.7) | 21.8 (20.1-23.6) | 22.1 (20.0-24.3) | 13.0 (11.7-14.6) | 10.0 (8.8-11.3) | 11.5 (10.3-12.9) | 10.4 (9.3-11.5) |
| Rangpur | - | - | - | 10.4 (9.4-11.4) | 9.4 (7.9-11.2) | 10.5 (9.3-11.5) | 11.1 (10.2-12.2) |
| Sylhet | 7.5 (6.6-8.6) | 8.9 (7.9-9.9) | 8.9 (7.4-10.6) | 7.6 (6.9-8.3) | 9.7 (7.2-12.9) | 7.9 (7.0-8.9) | 6.7 (6.1-7.4) |
