# Supplementary material for: Trends, district-level variations, and socioeconomic disparities in cesarean section delivery in Bangladesh
Source: PLoS One. 2025 Oct 31;20(10):e0334931. doi: 10.1371/journal.pone.0334931 (PMC12578250; doi:10.1371/journal.pone.0334931)
Supplement: S2 Table — (DOCX) [file pone.0334931.s002.docx]

**Supplementary Table 2. Multilevel mixed-effect logistic regressions assessing the associations between the survey years and overall CS delivery and CS delivery at the institution level in Bangladesh, adjusted for socio-demographic factors; BDHS, 1999/2000-2022 (n = 32,461)**

| **Characteristics** | **Overall CS delivery vs overall normal delivery,**  **aOR (95% CI)** | **Institutional CS delivery vs institutional normal delivery, aOR (95% CI)** |
| --- | --- | --- |
| **Survey years** |  |  |
| 1999-2000 (ref) | 1.00 | 1.00 |
| 2004 | 1.42 (1.11-1.82)*** | 1.47 (1.11-1.95)*** |
| 2007 | 2.64 (2.11-3.32)*** | 2.13 (1.64-2.78)*** |
| 2011 | 5.09 (4.13-6.27)*** | 3.10 (2.44-3.93)*** |
| 2014 | 7.10 (5.78-8.74)*** | 3.34 (2.64-4.23)*** |
| 2017-2018 | 11.26 ( 9.18-13.82)*** | 4.62 (3.66-5.83)*** |
| 2022 | 17.86 (14.57-21.89)*** | 5.34 (4.28-6.77)*** |
| **Mother’s age at birth** |  |  |
| **≤**19 years (ref) | 1.00 | 1.00 |
| 20-34 years | 1.53 (1.42-1.65)*** | 1.46 (1.32-1.61)*** |
| ≥35 years | 2.20 (1.81-2.67)*** | 1.98 (1.55-2.53)*** |
| **Mother’s education** |  |  |
| No education (ref) | 1.00 | 1.00 |
| Primary | 1.60 (1.37-1.86)*** | 1.15 (0.94-1.42) |
| Secondary | 3.75 (3.25-4.33)*** | 1.75 (1.45-2.12)*** |
| Higher | 9.69 (8.27-11.35)*** | 2.90 (2.36-3.56)*** |
| **Mother’s formal employment status** |  |  |
| Employed (ref) | 1.00 | 1.00 |
| Unemployed | 1.38 (1.27-1.49)*** | 1.17 (1.05-1.30)*** |
| **Parity** |  |  |
| 1-2 (ref) | 1.00 | 1.00 |
| >2 | 0.49 (0.45-0.53)*** | 0.64 (0.57-0.71)*** |
| **Child’s gender** |  |  |
| Male (ref) | 1.00 | 1.00 |
| Female | 0.86 (0.81-0.92)*** | 0.93 (0.86-1.01) |
| **Place of residence** |  |  |
| Urban (ref) | 1.00 | 1.00 |
| Rural | 0.52 (0.48-0.56)*** | 0.94 (0.86-1.03) |
| **Random effect^a^** |  |  |
| Cluster level variance across survey years (SE)^b^ | 0.21 (0.02) | 0.14 (0.02) |
| **Model summary** |  |  |
| Intra-class correlation Coefficient (ICC) | 6.12% | 4.07% |
| AIC | 25028.56 | 14027.53 |
| BIC | 25171.03 | 14152.02 |

**Note:** ***p<0.01, **p<0.05; aOR=adjusted Odds Ratio; CI: Confidence intervals; Ref: Reference group; AIC=Akaike’s Information Criterion; BIC = Bayesian Information Criteria; SE = Standard Error. Cluster level variance across survey years estimates of random effects are reported. ^b^Significance of random effects evaluated by comparing the model with a similar one in which random effects were constrained to zero.
