# Supplementary material for: Trends, district-level variations, and socioeconomic disparities in cesarean section delivery in Bangladesh
Source: PLoS One. 2025 Oct 31;20(10):e0334931. doi: 10.1371/journal.pone.0334931 (PMC12578250; doi:10.1371/journal.pone.0334931)
Supplement: S1 Fig — (DOCX) [file pone.0334931.s003.docx]

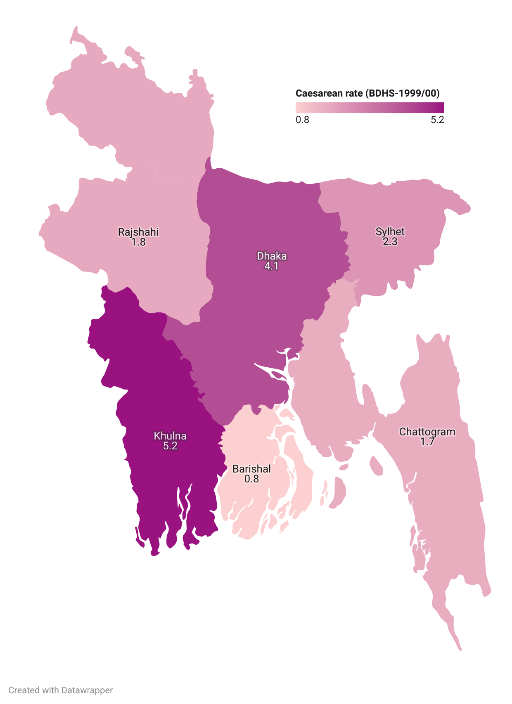

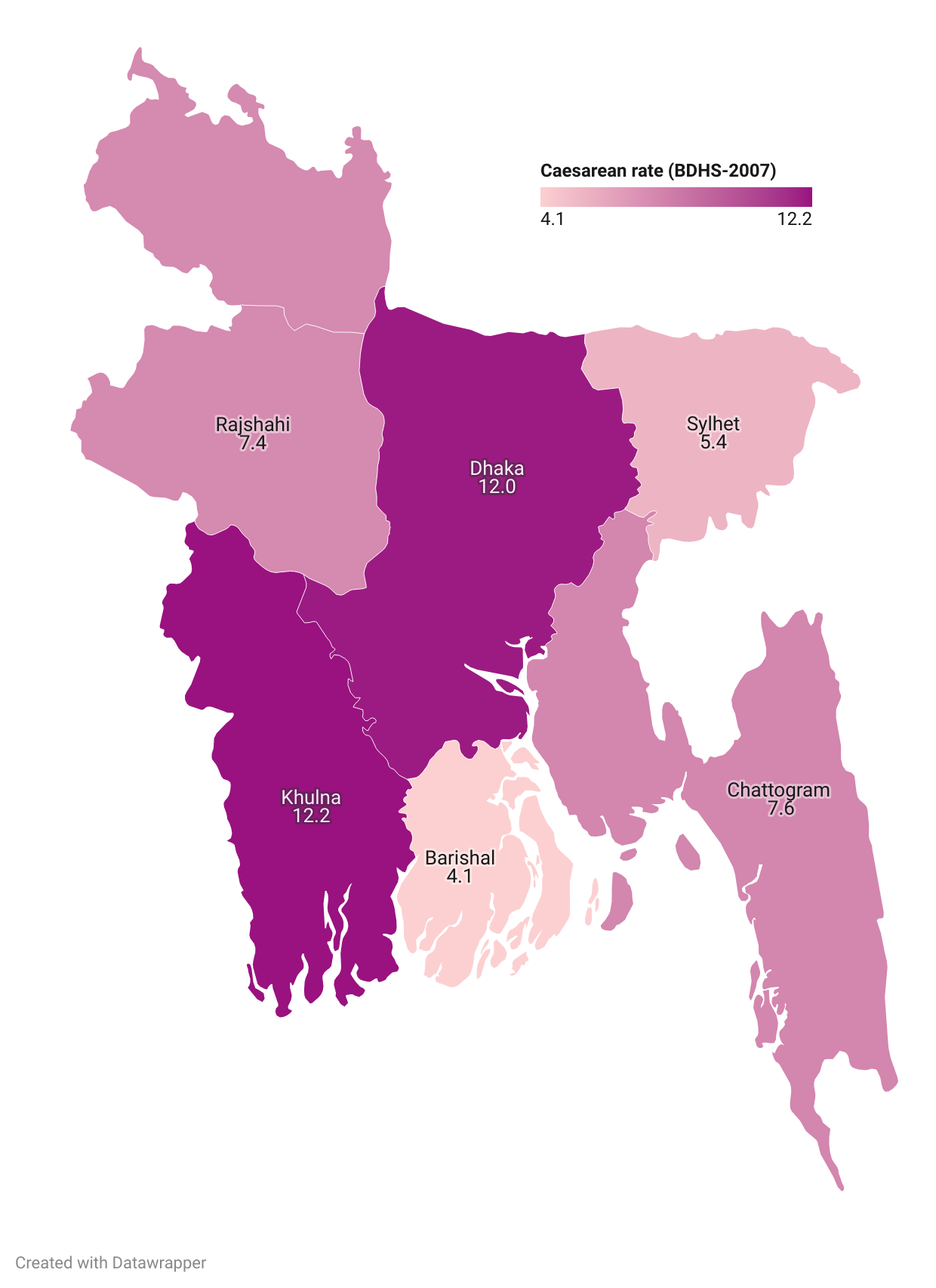

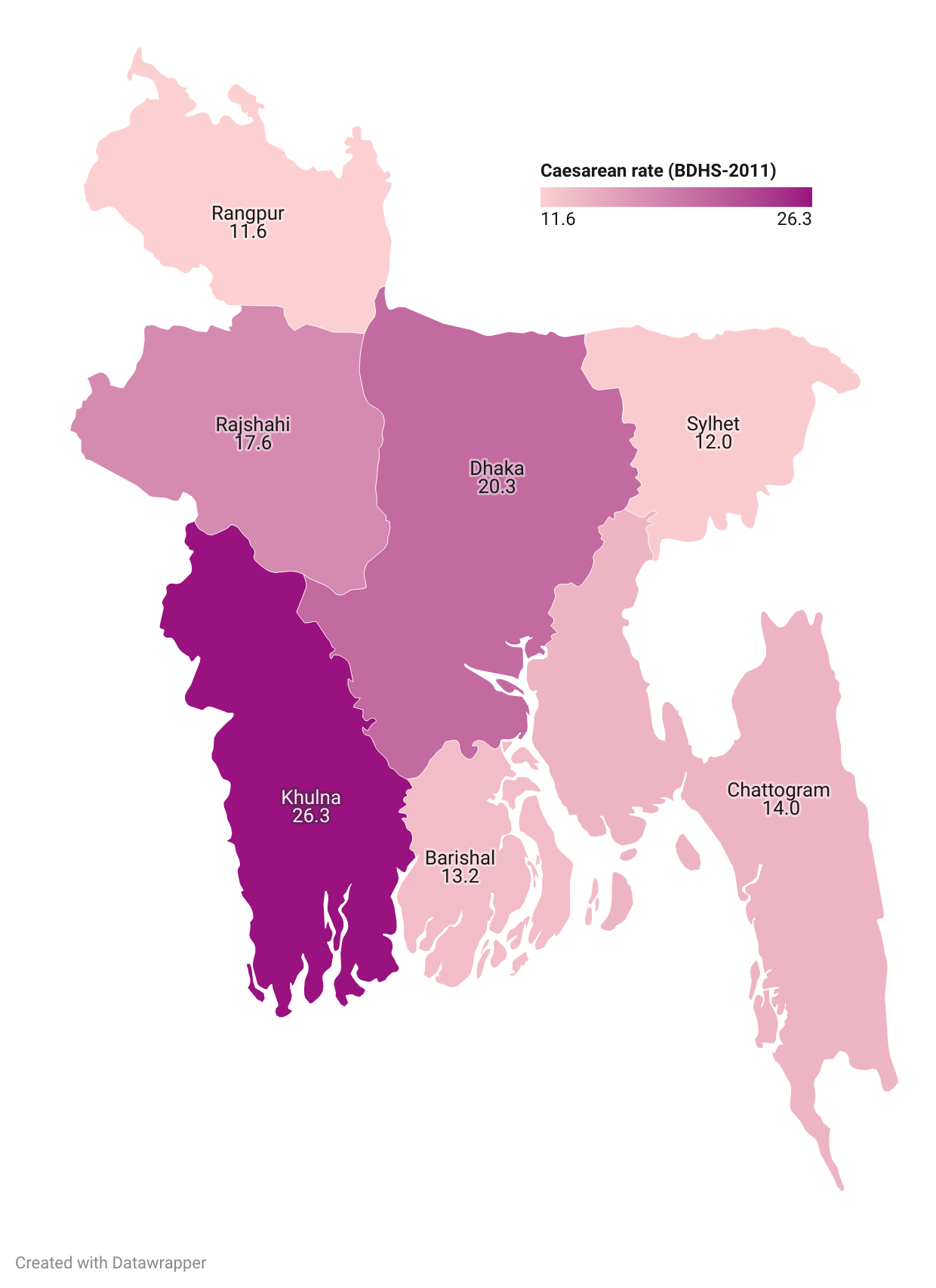

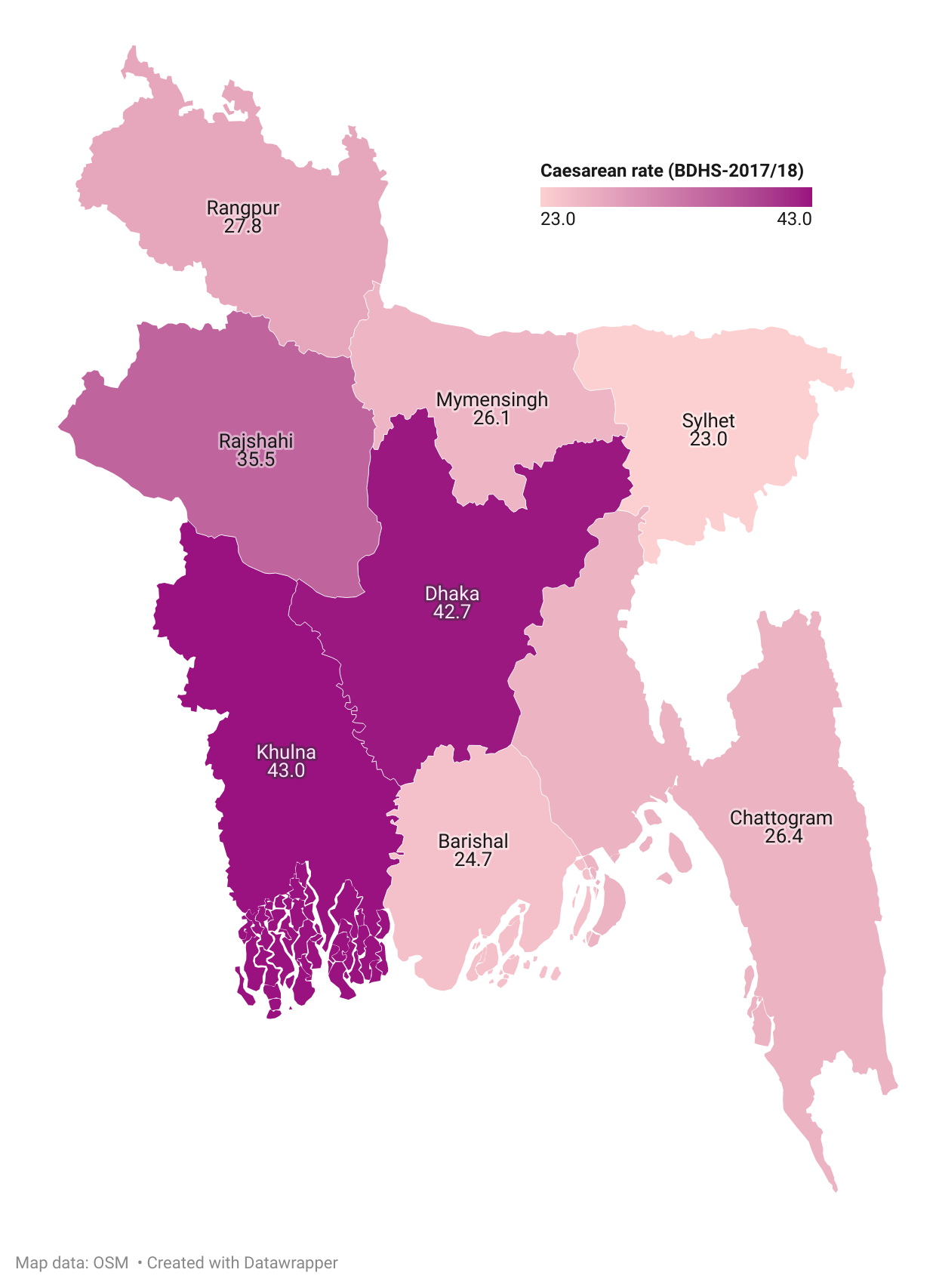

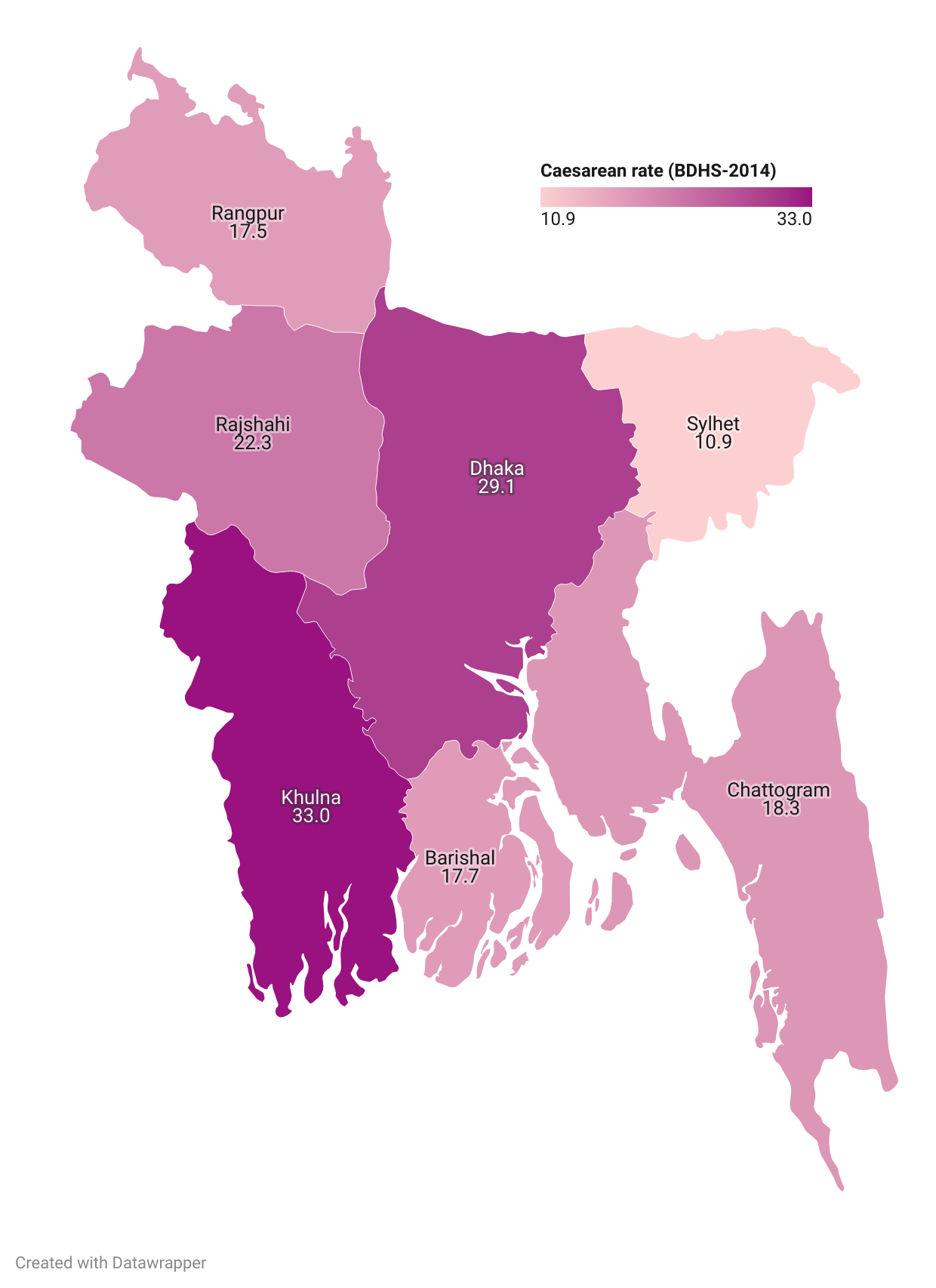

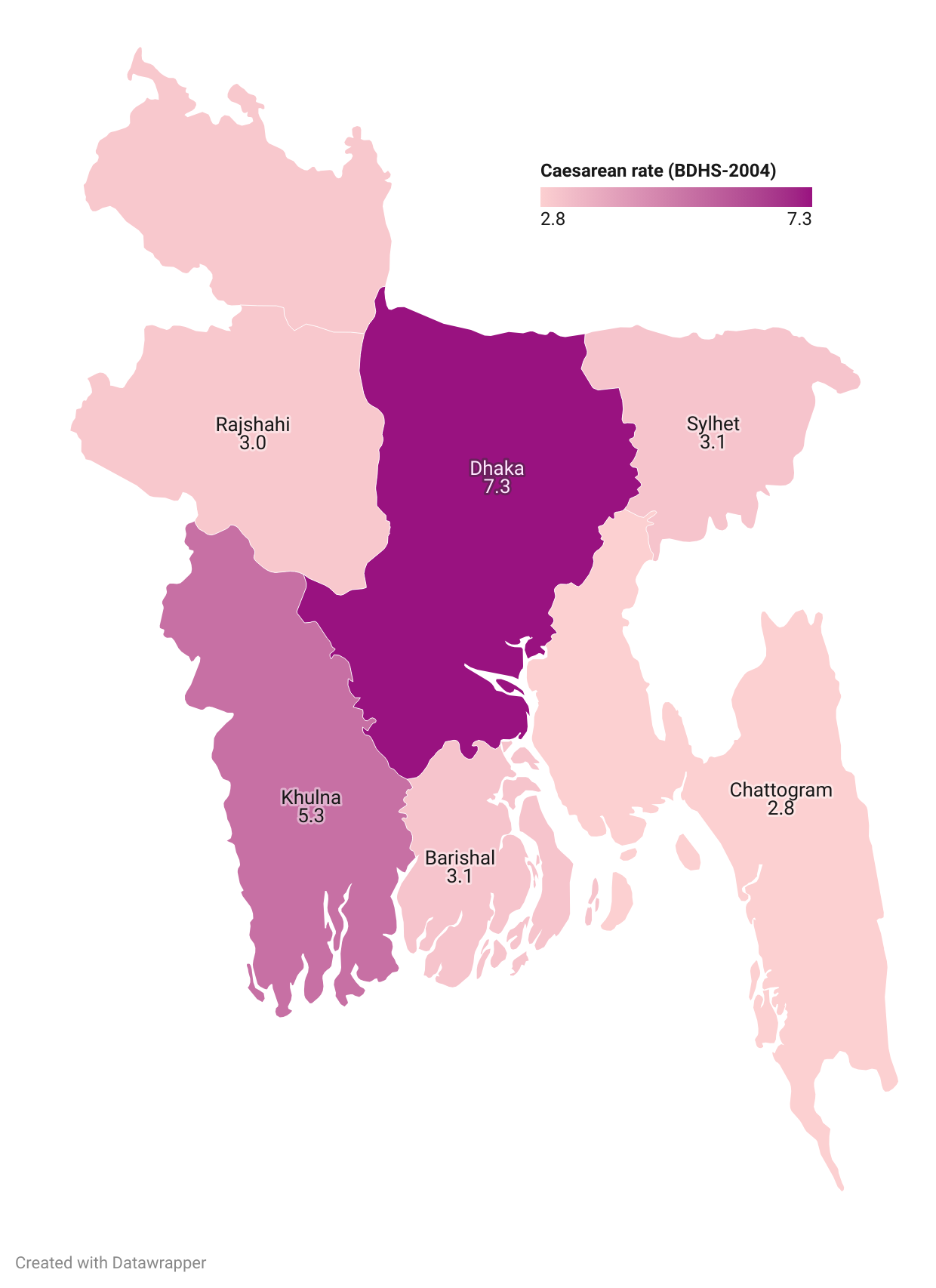

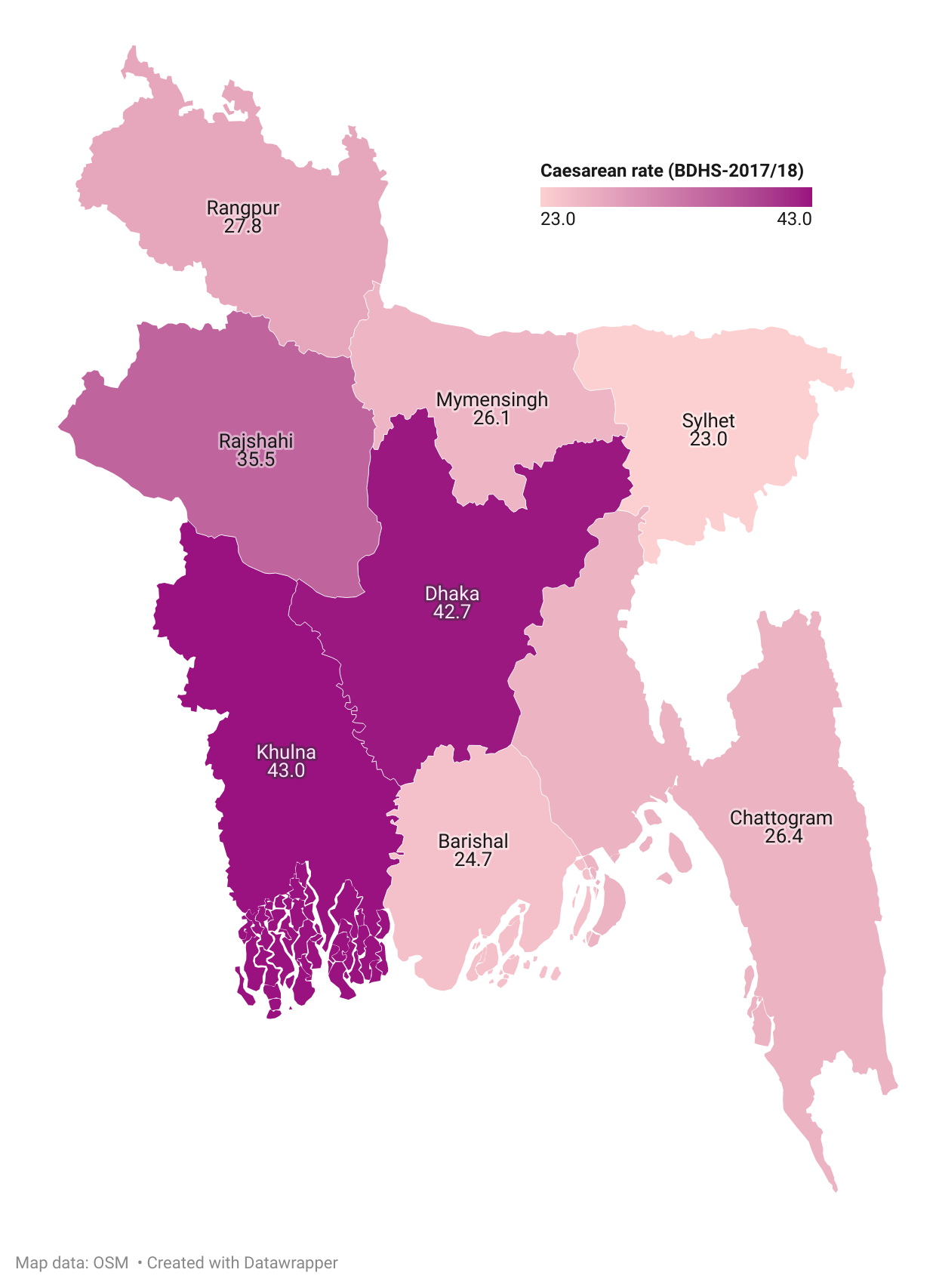


**Supplementary figure 1: Trend in cesarean sections delivery rates across divisions in Bangladesh from 1999/2000 to 2022**
