# Supplementary material for: Trends, district-level variations, and socioeconomic disparities in cesarean section delivery in Bangladesh
Source: PLoS One. 2025 Oct 31;20(10):e0334931. doi: 10.1371/journal.pone.0334931 (PMC12578250; doi:10.1371/journal.pone.0334931)
Supplement: S3 Fig — (DOCX) [file pone.0334931.s005.docx]

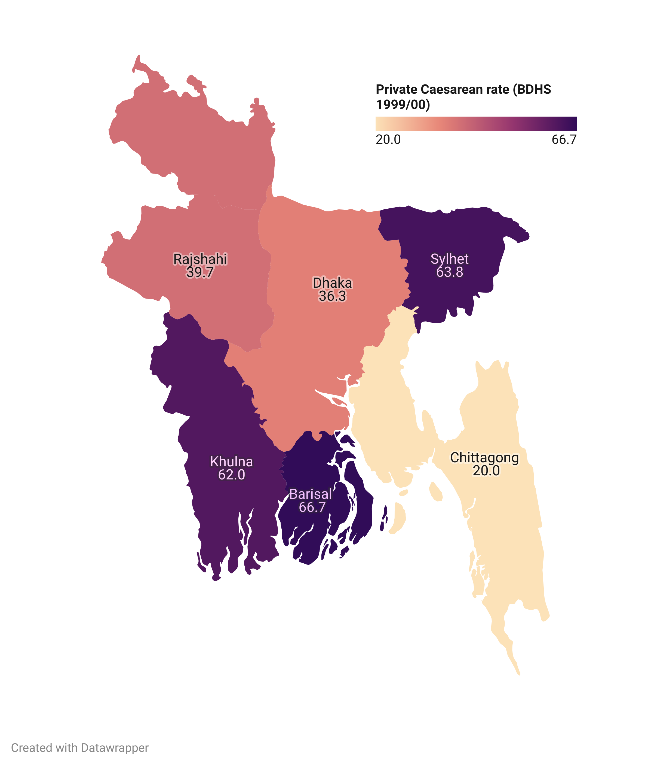

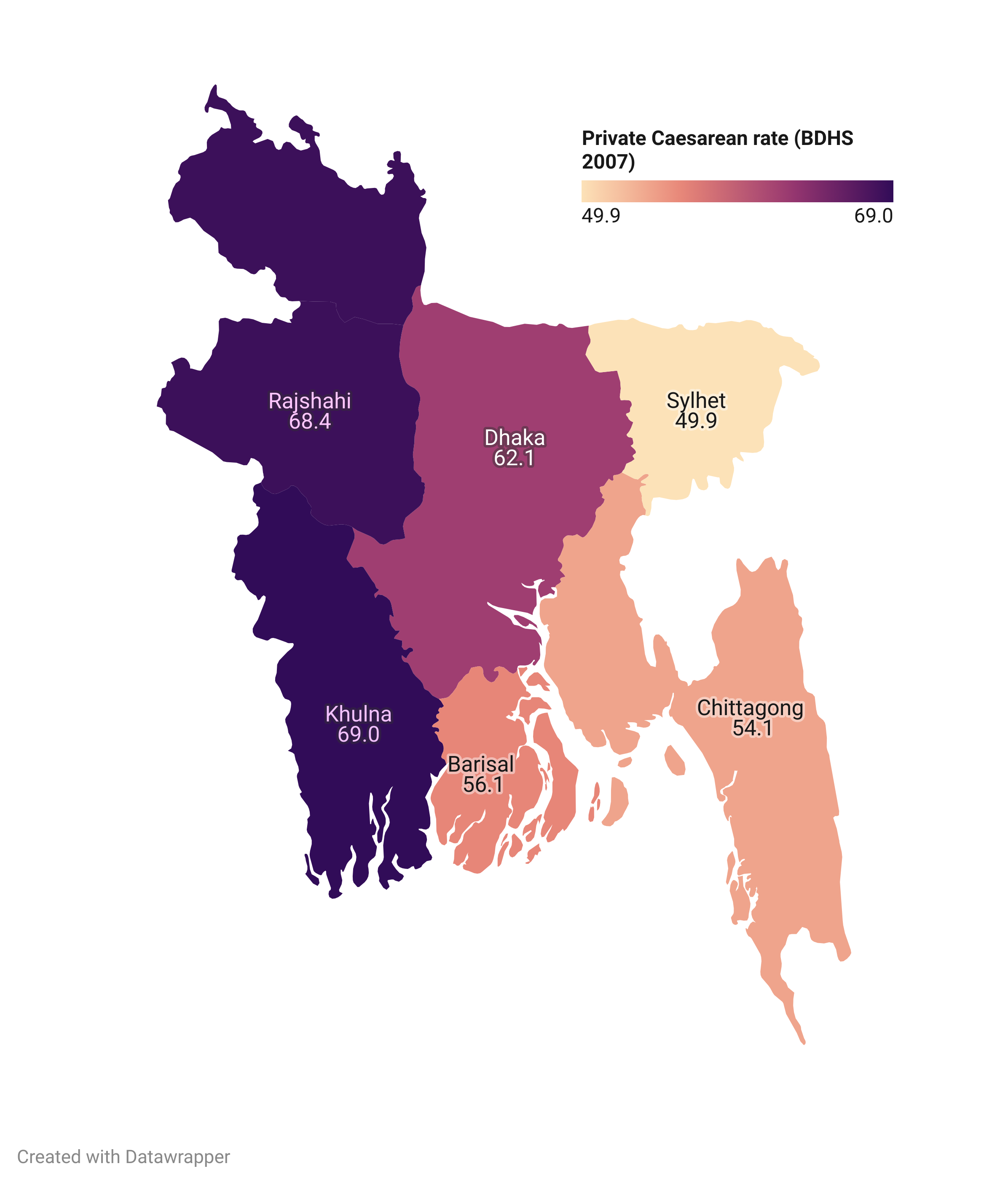

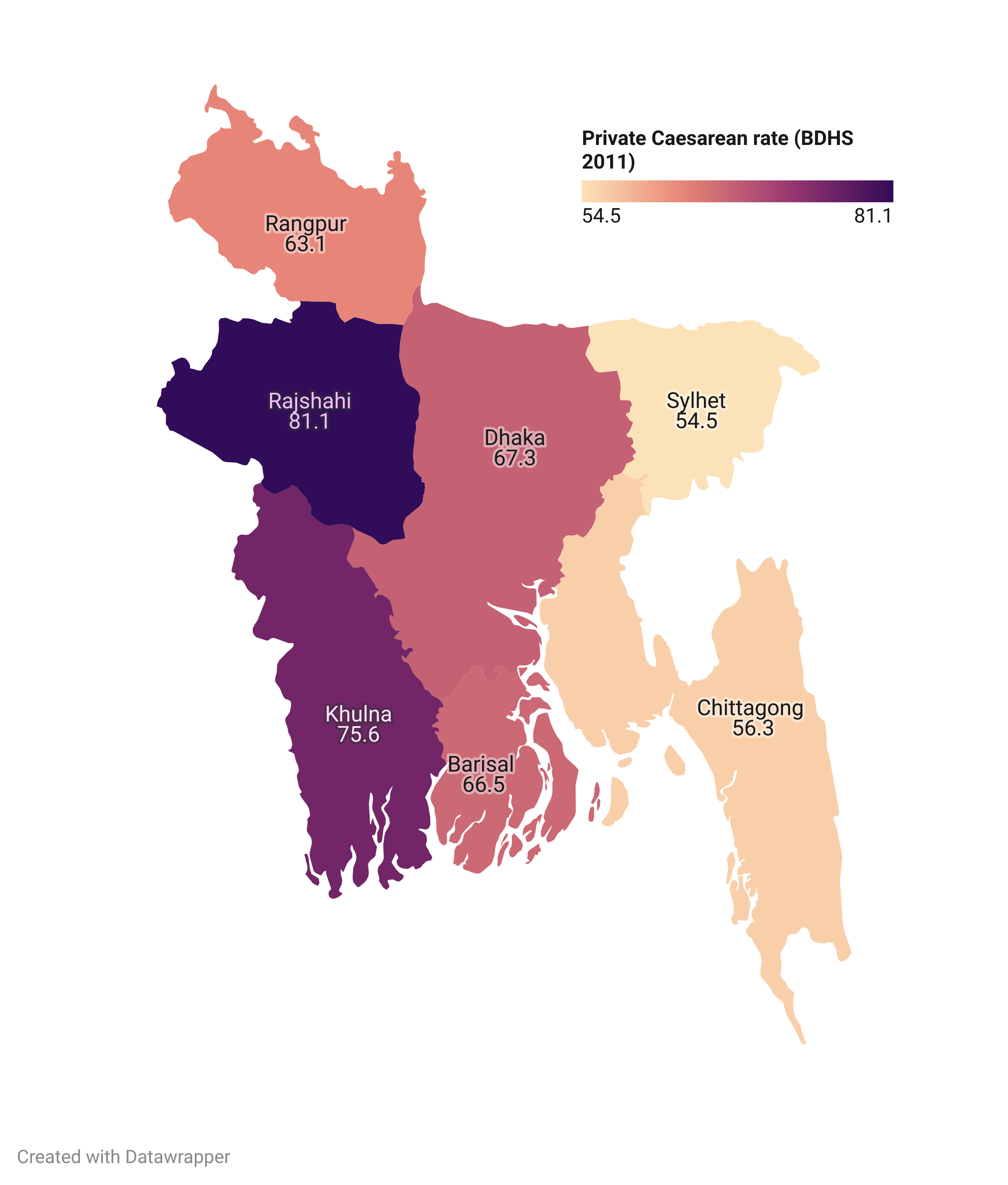

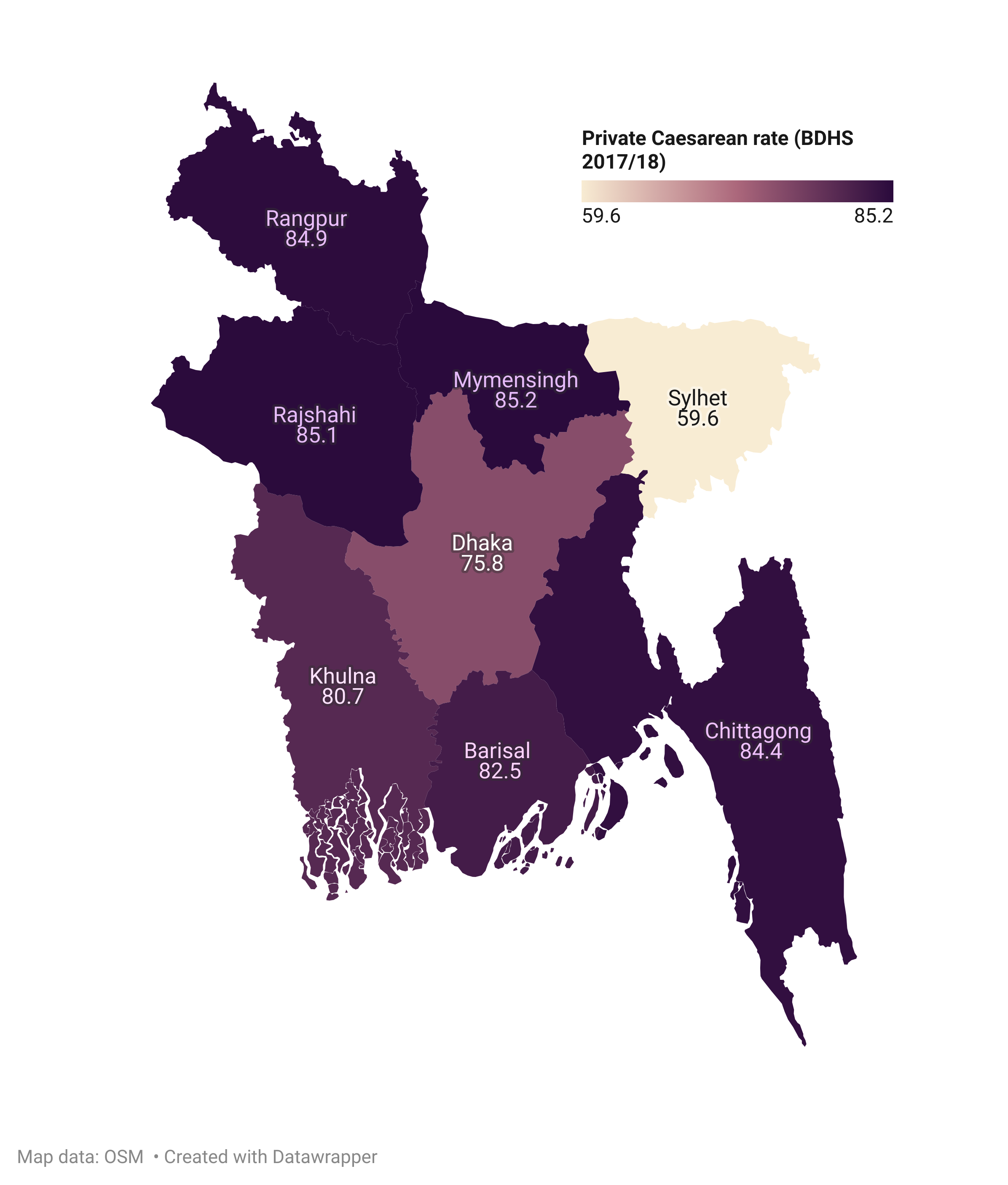

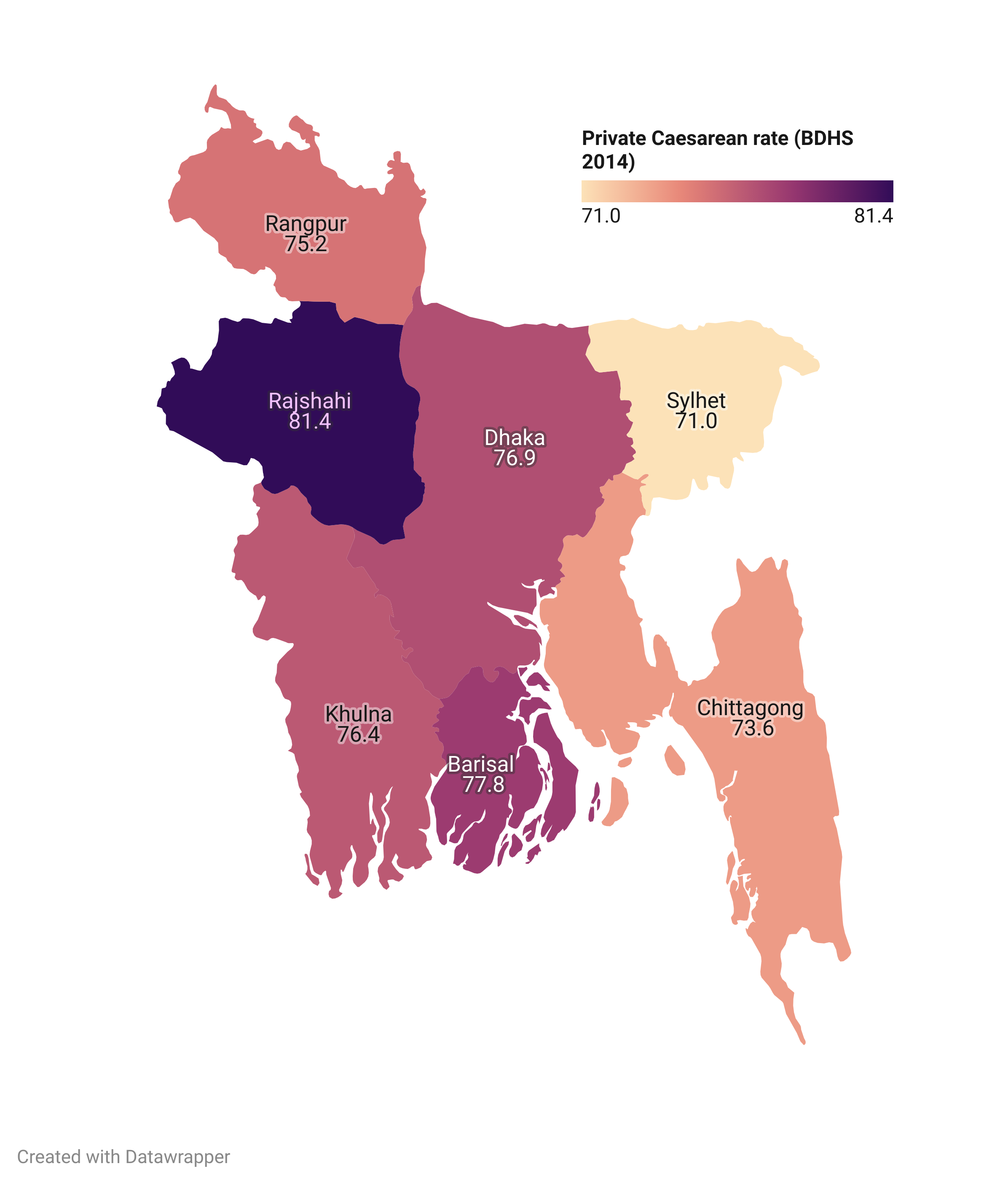

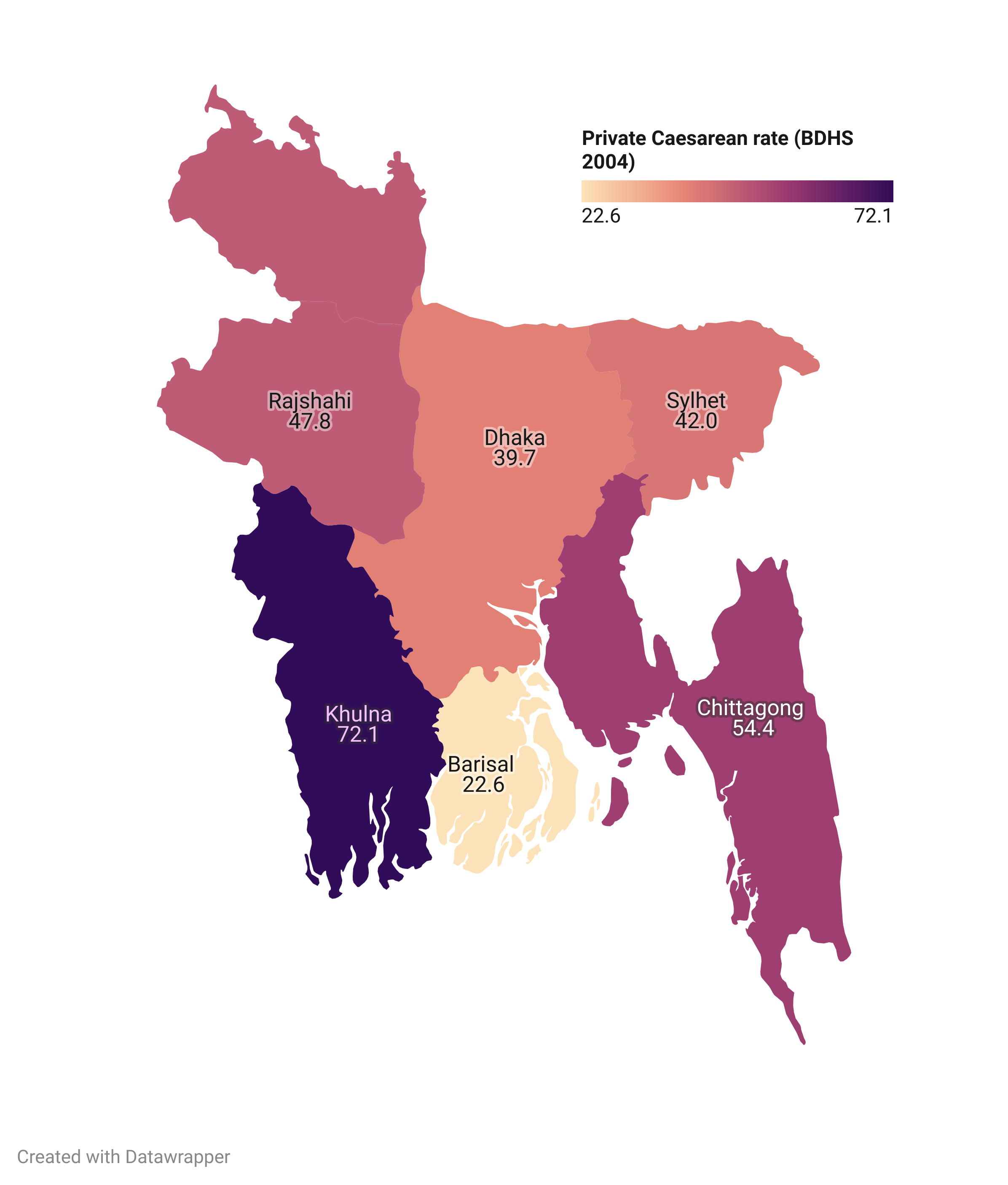

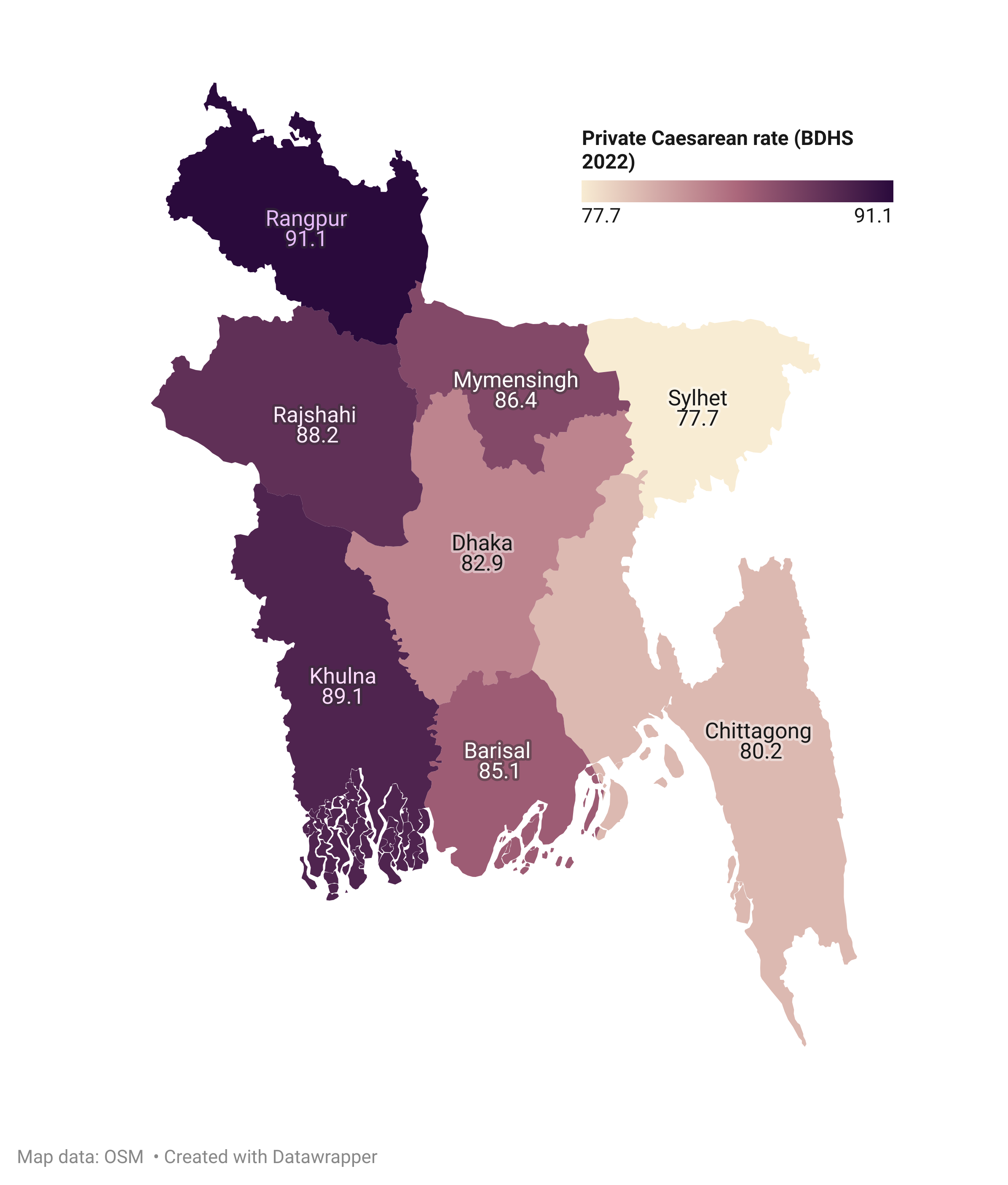


**Supplementary figure 3: Trend of cesarean section delivery rates performed in private healthcare facility across divisions in Bangladesh from 1999/2000 - 2022**
