# Supplementary material for: Trends, district-level variations, and socioeconomic disparities in cesarean section delivery in Bangladesh
Source: PLoS One. 2025 Oct 31;20(10):e0334931. doi: 10.1371/journal.pone.0334931 (PMC12578250; doi:10.1371/journal.pone.0334931)
Supplement: Attachment — Submitted filename: Response to Reviewers comments.docx [file pone.0334931.s006.docx]

**Authors’ responses to reviewers’ comments**

| **Reviewers’ comment** | **Authors’ response** |
| --- | --- |
| **Reviewer#1** |  |
| The authors studied trends, district-level variations, and socioeconomic disparities in cesarean section delivery and its association with neonatal mortality in Bangladesh. Some suggestions were listed as follows: | We thank you for minutely review our paper. We have addressed your comments and provided a point-by-point response below. |
| 1. In the Statistical Analysis Section, “We employed multilevel mixed-effect binary logistic regressions to investigate factors associated institutional delivery, CS delivery at the population level, and CS delivery at the institutional level.” Can you provide the statistical equation?  How can you explain CS delivery at the population level, since we know that the mixed-effect model is the individual level model? | We have now added statistical equation in the ‘Statistical Analysis’ section.  We have estimated the overall rates of CS delivery which was labeled “population level cesarean”. However, due to the confusion arisen for this term, we have now changed the term as overall CS cesarean delivery throughout the manuscript. Please see the revised version of the manuscript. |
| 2. In Table 3, some of the cell frequency is zero; this zero is observed rather than structural; this is observed zero. Could you offer us the confidence interval so we can use small sample inference to estimate the confidence interval? | Thank for your concern. We have now presented a figure across divisions instead of districts due to lower sample size in several districts. No required changes to address this comment. |
| 3. What is the interpretation of R^2=0.2286 in Figure 1? | We have included the interpretation of R^2=0.2534 (currently presented in figure 2) in “Socio-economic differentials of cesarean delivery across divisions” section which now reads-  *…………………………………….* *Substantial variations in the risk ratios of CS delivery between the poorest and richest quintiles were observed across districts, ranging from less than 2.0 (in Narail, Chapai Nawabganj, Comilla, Jhenaidah, Rajshahi, Chuadanga, Satkhira, Bagerhat, Narsingdi, Kushtia, Manikganj, and Jhalokati) to 8.6 (in Sunamganj) (Figure 2). Thus, higher CS delivery rates corresponded to greater relative disparities, with an R² value of 0.2534.* |
| 4. In Table 4 and Table 5, the authors presented the results of mixed effect models. Can you clearly specify what the random effect terms are? Have you only used random intercepts? Could you please provide the value and interpretation of the standard error of random effect terms? | Many thanks. We have included cluster and survey years-as random effect terms. We have also provided the value and interpretation of the standard error of random effect terms in Table 4 (cluster level) and Supplementary Table 3. Please see the updated version of the manuscript. |
| 5. How did the mixed effect approaches address changes in CS delivery rate during survey years? | We have now run a separate model, considering the year of the survey as an exposure variable to address this comment, and the relevant results are presented in Supplementary Table 3. |
| 6. How did the mixed effect approaches address changes in neonatal mortality rate during survey years? | As part of the comprehensive revision, we have deleted neonatal mortality related results. |
